# On Dislocations of the Astragalus

**Published:** 1844-10

**Authors:** 


### PART FOURTH.

### Medical Intelligence.

### ON DISLOCATIONS OF THE ASTRAGALUS.

### REPLY TO MR. TURNER'S RECLAMATION, PUBLISHED IN THE PROVINCIAL MEDICAL AND SURGICAL JOURNAL, AUG. 7, 1844.

In the last Number of the British and Foreign Medical Review, we noticed Mr. Turner's paper on "Dislocations of the Astragalus," in the ordinary discharge of our duties towards our readers. Our observations have appeared to Mr. Turner, hypercritical, uncourteous, unfriendly, unfair, and unjust, and he has consequently published in the 'Provincial Medical and Surgical Journal,' of the 7th of last August, a letter addressed to Dr. Hastings, in which he endeavours to establish these grave charges. Though the imputation of deliberate unfairness is calculated to excite some indignation, our consciousness that the charge is utterly groundless, completely allays any such feeling. Had we, through inadvertence or negligence, unintentionally aggrieved Mr. Turner, we would feel pride and pleasure in frankly acknowledging our error, and we shall proceed calmly and dispassionately to inquire whether we are really chargeable with mistake.

Mr. Turner's first tangible charge is, that we endeavoured to make it appear that he has not done justice to Sir A. Cooper's work on 'Dislocations and Fractures of the Joints.' Mr. Turner is mistaken; we simply averred that he had not availed himself of all the materials applicable to his purpose, contained in that work. If that averment can be interpreted into a charge of injustice, and assuredly it cannot, Mr. Turner has been unjust to himself and not to Sir A. Cooper. The question, however, whether Mr. Turner did or did not omit relevant cases, is a question of fact which we shall presently examine; but we must previously advert to an alleged unwillingness on our part to admit the truth of Mr. Turner's opinion respecting the comparative frequency of simple and compound dislocations of the astragalus: on Mr. Turner's own showing, we have manifested this unwillingness in a very singular manner, for, to use his own words, the reviewer "*confirms*" his opinion "*by additional evidence.*" We might here congratulate ourselves on this admission, that we have added something to the undoubtedly valuable materials collected by Mr. Turner; but letting that pass, we have, it is alleged, been guilty of "servile adherence to authority," in venturing to add that "it remains to be seen, however, whether Sir A. Cooper's opinion may not prove true, if ever a very extensive series of cases is collected." We beg to assure Mr. Turner that we are not slaves either to authority, prejudice, or passion; we are willing to yield implicit obedience to facts, when they are sufficiently numerous to warrant us in deducing from them a definite opinion. So far from servilely adopting Sir A. Cooper's dictum, we have contributed "additional evidence" against its truth; we would much rather be right with Mr. Turner than wrong with Sir A. Cooper; but we still venture to retain our conviction, that statistical conclusions are eminently uncertain, unless they are deduced from a very large number of observations—unless all, or nearly all, the ascertainable facts capable of influencing the conclusion are compared; and it is in obedience to this universally admitted rule of statistical investigation, that while we provisionally admit Mr. Turner's opinion, we hold that the comparison of a very extended series of cases may possibly ultimately disprove it. How the result of a numerical fact can be influenced one way or the other by its having been elicited by the researches of a "provincial hospital surgeon," we confess our inability to understand, and must therefore leave Mr. Turner's observation on that head unanswered.

We have now to deal with the specific question, whether Mr. Turner was right or wrong in omitting from his statistical tables certain alleged cases of dislocation of the astragalus, contained in Mr. B. Cooper's edition of Sir A. Cooper's work, on 'Dislocations and Factures of the Joints.' Mr. Turner admits that he omitted the cases we referred to, and justifies the omission by certain reasons assigned, which it is, of course, our duty to examine; and in so doing, it will, to use Mr. Turner's words, "save time and trouble" to inquire "what is really meant by dislocation of the astragalus;" a point, respecting which, Mr. Turner says, we require information, and although he "may be considered presumptuous in attempting it," he shall endeavour to set us right.

Mr. Turner's definition of a dislocation of the astragalus is this, that *all* the articular surfaces of the bone must be, in some degree, displaced, and consequently, that "if the astragalus retain its natural attachments with the tibia and fibula, but happen to be torn from the os naviculare, or os calcis, or both, it would not be a dislocation of the astragalus, but a diastasis, or disjunction of one, or both of these bones *from* the astragalus. In a word, so long as this bone is in situ, in reference to either of the bones of the leg, or those of the foot with which it is articulated, the astragalus *itself* is not dislocated. . . . . . . A bone that has been dislocated must, to constitute this accident, be ex situ in reference to *all* its articular surfaces, however numerous the articular surfaces may be; so long, therefore, as the astragalus is not severed from the tibia, fibula, os calcis, and os naviculare, or is retained in natural anatomical contact with either of them, the dislocation is of the bone or bones in connexion with it, not of the astragalus itself." Now this language is very precise, admits of no mistake. If we were so hypercritical as Mr. Turner supposes, we might comment on the strange consequences that must of necessity flow from the new doctrine—"*A bone that has been dislocated must, to constitute this accident, be ex situ in reference to* ALL *its articular surfaces, however numerous the articular surfaces may be*"—if we attempt to apply it to dislocations of the long bones: and we might, without the remotest approach to hypercriticism, insist that Mr. Turner means it to be so applied, as a few lines above the last extract he maintains, that according to his explanation, "our views of dislocation become definite, not only with respect to the accidents occurring to the astragalus, but in reference to *every other kind* of dislocation." Let us, however, limit the doctrine to the astragalus; and even then we maintain that Mr. Turner is wrong, and that partial or complete disconnexion of the astragalus from the bones of the leg, is *not* essential to constitute a dislocation of the astragalus; and in support of this assertion, we need only appeal to the whole stream of authorities—J. L. Petit, Boyer, Moufalcon, Savary, Marjolin, A. Cooper, Sanson, Laugier, Roux, &c. But perhaps it may be said—here again is servility to authority; and if so, we reply, that two points have to be considered in a question of nomenclature: 1st. What the received nomenclature *is;* 2, what it *ought* to be. The first point can only be decided by an appeal to usage, that is to authority; and we repeat that the whole weight of authority is with us and against Mr. Turner; for Rognetta alone—the exception that proves the rule—sanctions Mr. Turner's view; and even Rognetta somewhat inconsistently adds, "the essential condition for luxation of the astragalus is, that the head of that bone has left the eliptical cavity of the scaphoid bone." As to the 2d point, viz., what the nomenclature *ought* to be, we are of course dispensed from the necessity of considering that question. We had no choice in our review, save to adhere to recognized usage; had we done otherwise, Mr. Turner would indeed have had cause for complaint; but thus much we must say, that not merely the entire weight of authority, but the whole weight of argument preponderates against Mr. Turner's, or rather, Rognetta's view. Let any impartial inquirer examine Rognetta's own account of the mechanism of anterior dislocations of the astragalus, either in our review of Mr. Turner's paper, or, better still, in the original memoir, and he will have the key to the argument, which irresistibly proves the propriety of the received nomenclature; neither time, nor space, nor fitness of occasion, allows us to enter fully into this branch of the subject, on which we have indeed said enough; but we cannot refrain from remarking, that Mr. Turner violates his own doctrine, so explicitly laid down in the extract above given. He quotes Boyer's last case as an example of dislocation of the astragalus, and actually founds one of his practical inferences on the result of this very case, and yet, according to Mr. Turner's views, this case is *not* one of dislocation of the astragalus, as that bone was not separated from its connexion with the bones of the leg. We have, therefore, here the satisfaction of appealing in our justification to Mr. Turner himself. We shall not retort the charge of ignorance as to what is really meant by dislocations of the astragalus: we should be ashamed to throw such an imputation on Mr. Turner, whom we unfeignedly believe to be a gentleman as eminent in professional attainments, as he is respectable in private character. We are willing to believe that Mr. Turner, in consequence of writing in some hurry, and with more excitement, fell into an inadvertence. It may be, indeed, that Mr. Turner deliberately adopts Rognetta's very peculiar view; but if so, we must be pardoned for remarking, that he should have explicitly declared his deviation from the received opinion in his essay, and should, in his letter to Dr. Hastings, have abstained from imputing ignorance to his reviewer for adhering to known usage.

With respect to the cases in Sir A. Cooper's work, omitted by Mr. Turner: cases 193 and 200 were omitted, we are told, because the connexions of the astragalus with the tibia and fibula remained undisturbed. We have already disposed of that point. Case 192, it is said, is doubtful, but Mr. Hey and Sir A. Cooper both expressly recognize it as a "compound luxation of the astragalus." Case 191, Mr. Turner says, cannot be termed dislocation of the astragalus; and on reconsideration, we admit that, properly speaking, it cannot, but its bearing on the history of the accident is sufficiently important to induce Sir A. Cooper to again notice it under that head, after having previously given the case more fully. Case 194, Mr. Turner informs us, he rejected, as being an example of compound luxation of the tarsal bones; but the prominent fact mentioned in the case is, that the head of the astragalus was displaced, and, therefore, we rank it among dislocations of that bone. Case 196 was passed by because there was comminuted fracture as well as dislocation of the astragalus. But need we remark that fracture is by no means an unusual complication of dislocation of the astragalus, and coexisted in several of the cases adopted by Mr. Turner himself.

Mr. Turner complains that our comments on his selections from Boyer's cases are hypercritical. We expressly said, that the inaccuracy respecting Deniel's case was trifling. Had it stood alone, it would have been indeed hypercritical to notice it; but our argument was a cumulative one, and, therefore, our observation on the case was justifiable as a member of that argument. We are accused of wrongly saying, that Boyer's work is not referred to for that case, but we of course only meant that the specific reference thereto to enable the case to be identified was not given, as sufficiently appears from our previous remark that the case is given as having occurred in Boyer's practice. We are also reproached with having noticed the oversight of Boyer's case of dislocation of the foot, being quoted as one of dislocation of the astragalus backwards, and then commenting on the different view taken of "*these* cases" by Boyer and Mr. Turner, while we were well aware that they "were writing on two different accidents," and that, therefore, "there was no difference of opinion between" them, and that they were both correct in their remarks on the subjects on which they respectively wrote. We acknowledge the dexterity, but we say nothing of the candour of putting the matter in this way. Undoubtedly we were well aware that Boyer and Mr. Turner were writing on two different accidents, for that was the very oversight we pointed out, but they were writing about *one and the same case*, and we adverted to the difference of their views respecting *that case*. Mr. Turner pleads that the omission of the case altogether "would not have detracted, in the slightest degree, from his practical argument;" undoubtedly it would not, as it does not in the slightest degree bear on that argument; but we ask, would not the omission of the case have added something to his accuracy? As to Dupuytren's cases, we neither doubted nor implied any doubt, that twelve cases of dislocation of the astragalus occurred in his practice; we are well aware of the references for that fact given by Mr. Turner, and could point out others to him; but we said, and we repeat, that only five of them are distinctly recorded, and, consequently, to those five alone did we refer, while Mr. Turner quotes but three of them. But we are further charged with hypercriticism, mystification, and injustice, in our comments on Mr. Turner's inaccuracies respecting Dupuytren's cases. We shall content ourselves with asking—was it hypercritical or unjust to observe, that in a table purporting to give the statistical results of the treatment of dislocations of the astragalus, one of Dupuytren's cases is enumerated among those headed "bone allowed to remain in its new situation," whereas, in point of fact, the bone was *excised;* and that another case, placed in the same class, should have been placed under the head "partial reduction." Mr. Turner says, he cannot understand this latter distinction; perhaps not; but if so, why did he adopt the distinct class "partial reduction" in his statistical classification of the treatment and results of cases?

Mr. Turner offers no explanation why he omitted two of Dupuytren's cases. Three of Desault's he did not specially notice, as they were complicated with compound dislocation of the ankle-joint, a circumstance which however, in our humble judgment, greatly enhances their importance; the other four are not alluded to. Hey's cases were omitted, as they are not contained in the first edition of that eminent surgeon's work; we have been ourselves too often embarrassed from a similar cause not to fully admit the validity of this plea.

We have now to deal with by far the gravest imputation brought against us by Mr. Turner,—an imputation affecting our honesty and fairness. It is said that we "selected all the vulnerable parts of the paper," and "*sedulously* avoided the mention of any points of merit." If such were the case, we would indeed feel disgraced; but we firmly, though we trust temperately, deny the accusation. We are accused of not having noticed the chapters on the anatomical relations of the astragalus, and on the forces which produce its dislocations; but does Mr. Turner forget that a portion of our article is devoted to those very considerations in our notice of Rognetta's memoir? Was not our silence as to Mr. Turner's observations on these points sufficiently significant why we were silent? But we are now compelled to say what we then abstained from saying, that we—it may be erroneously, but most assuredly in the best exercise of our judgment, *quantum valeat*—considered and still consider Rognetta's investigations in this respect so greatly superior to Mr. Turner's speculations, that we deemed it no injustice to our readers, and far from unkindness to Mr. Turner, to pass those parts of his paper in silence. Mr. Turner is of opinion, that "we cannot, by any experiment, illustrate or imitate the mechanism of dislocation." (p. 22.) And so long as he retains this opinion, we fear he can scarcely so write on the subject as to satisfy the exigences of modern science. We regret, however, that even where we have awarded what we thought to be due praise, Mr. Turner is dissatisfied. He seems displeased at our saying that his principles of treatment are "on the whole very judicious," though we added, with the courtesy which we felt was due on practical points to a surgeon of his experience and capacity, that where we differed from him it was perhaps erroneously. Mr. Turner moreover accuses us of a "*perversion*" of one of his practical rules. We shall insert the original text, followed by our version of it, and then leave our readers to judge on whom "*perversion*" is justly chargeable.

"*If the astralagus be* PARTIALLY DISLOCATED, *and not twisted round* (*as is often the case when the dislocation is complete,*) *there is reason to hope that reduction may be accomplished.*" (p. 89.)

"Reduction should always be accomplished in partial and simple dislocation of the astragalus; *but if it is impeded by the astragalus being twisted, etc. operative interference,*" *etc.*

The words printed in italics in the latter extract are suppressed by Mr. Turner. We make no comment on this. Had we been influenced by the malevolent motives attributed to us, might we not have made out a plausible and in many respects a real case, showing that Mr. Turner had been anticipated in his practical views by Laugier, in his excellent paper on dislocations of the astragalus? but we felt that to do so would have been unjust, because we were persuaded that Mr. Turner had borrowed nothing from Laugier; that their points of agreement were but coincidences; and moreover that Mr. Turner had added many valuable practical points, which we took good care to mention in our article, carefully avoiding anything that would seem to question the originality which we thought due to him.

We think we have satisfactorily shown that we were not influenced by any unfair or unfriendly spirit in drawing up our review of Mr. Turner's essay. Indeed Mr. Turner himself impliedly acknowledges the justice of the *tenor* of our remarks, by pleading that his paper was hastily drawn up: but how could we go behind the author's

LIBRARY OF THE
Orleans Parish Medical Society explicit statement that "with a view to correct statistics," he had "made diligent search for published as well as unpublished cases," and given "all the cases" which, "after much labour," he had "been able to collect?" We consequently dealt with Mr. Turner's paper as a statistical one, and ought not to have done otherwise. This is the obvious, the unanswerable reason, why our article assumed the shape it did; and hence also the answer to the complaint that we reckoned Mr. Turner's cases as 44 in all, 16 being unedited. We did so because these cases alone are numbered by Mr. Turner, and these alone are introduced into his statistical tables; and no loophole for complaint is left there to Mr. Turner, for the cases which he does not number, but only mentions *incidentally*, are noticed by us as being *so* mentioned. Mr. Turner asks, "Is the Reviewer serious in recommending me to publish this essay?" That is not exactly our recommendation: but we were and are of opinion that he may convert it into an extremely valuable monograph by remedying certain defects which we attempted to point out. We saw enough in Mr. Turner's paper to induce us to think that he was capable of discharging his task in a manner worthy of his subject and highly creditable to himself. We expressed that opinion in our review, and by that opinion we abide. We are glad to learn that Mr. Turner, though uninfluenced by our advice, is yet induced by other motives to act in accordance with it; and we are gratified that Mr. Turner, despite the asperity of controversy, frankly acknowledges himself obliged to our humble labours for indicating materials which may prove useful to him in the execution of his laudable design.

*August, 11th,* 1844.

